# Bacterial Factors Associated with Lethal Outcome of Enteropathogenic *Escherichia coli* Infection: Genomic Case-Control Studies

**DOI:** 10.1371/journal.pntd.0003791

**Published:** 2015-05-15

**Authors:** Michael S. Donnenberg, Tracy H. Hazen, Tamer H. Farag, Sandra Panchalingam, Martin Antonio, Anowar Hossain, Inacio Mandomando, John Benjamin Ochieng, Thandavarayan Ramamurthy, Boubou Tamboura, Anita Zaidi, Myron M. Levine, Karen Kotloff, David A. Rasko, James P. Nataro

**Affiliations:** 1 Department of Medicine, University of Maryland School of Medicine, Baltimore, Maryland, United States of America; 2 Institute for Genome Sciences, Department of Microbiology and Immunology, University of Maryland School of Medicine, Baltimore, Maryland, United States of America; 3 Center for Vaccine Development, University of Maryland School of Medicine, Baltimore, Maryland, United States of America; 4 Medical Research Council, Fajara, The Gambia; 5 International Center for Diarrhoeal Disease Research, Dhaka, Bangladesh; 6 Centro de Investigacao em Saude de Manhica, Maputo, Mozambique; 7 Kenya Medical Research Institute/Center for Disease Control, Kisumu, Kenya; 8 National Institute of Cholera and Enteric Diseases, Kolkata, India; 9 Center for Vaccine Development—Mali, Bamako, Mali; 10 The Aga Khan University, Karachi, Pakistan; 11 Department of Pediatrics, University of Virginia School of Medicine, Charlottesville, Virginia, United States of America; Oxford University Clinical Research Unit, VIETNAM

## Abstract

**Background:**

Typical enteropathogenic *Escherichia coli* (tEPEC) strains were associated with mortality in the Global Enteric Multicenter Study (GEMS). Genetic differences in tEPEC strains could underlie some of the variability in clinical outcome.

**Methods:**

We produced draft genome sequences of all available tEPEC strains from GEMS lethal infections (LIs) and of closely matched EPEC strains from GEMS subjects with non-lethal symptomatic infections (NSIs) and asymptomatic infections (AIs) to identify gene clusters (potential protein encoding sequences sharing ≥90% nucleotide sequence identity) associated with lethality.

**Results:**

Among 14,412 gene clusters identified, the presence or absence of 392 was associated with clinical outcome. As expected, more gene clusters were associated with LI versus AI than LI versus NSI. The gene clusters more prevalent in strains from LI than those from NSI and AI included those encoding proteins involved in O-antigen biogenesis, while clusters encoding type 3 secretion effectors EspJ and OspB were among those more prevalent in strains from non-lethal infections. One gene cluster encoding a variant of an NleG ubiquitin ligase was associated with LI versus AI, while two other *nleG* clusters had the opposite association. Similar associations were found for two *nleG* gene clusters in an additional, larger sample of NSI and AI GEMS strains.

**Conclusions:**

Particular genes are associated with lethal tEPEC infections. Further study of these factors holds potential to unravel the mechanisms underlying severe disease and to prevent adverse outcomes.

## Author Summary

Typical enteropathogenic *E*. *coli* (tEPEC) strains are associated with high mortality among infants with moderate-to-severe diarrhea, but most infants infected with tEPEC strains survive, and some have no symptoms. To investigate the bacterial factors associated with severe outcome, we determined the genomic sequences of 70 EPEC strains. Twenty four tEPEC strains came from children with lethal infections. The prevalence of each gene was compared to that in strains from 23 matched infants who had non-lethal symptomatic infection and to that in 23 matched infants who had asymptomatic infection. We identified 392 genes associated with outcome, some of which were more prevalent in strains from lethal infections, while others were less prevalent. The genes included several encoding potential virulence factors such as type 3 secreted effectors and enzymes involved in O-antigen synthesis. A PCR assay validated the association of groups of alleles encoding variants of the NleG ubiquitin ligase with clinical outcome. Further study of the factors associated with severe outcome could lead to novel diagnostic, therapeutic and prevention strategies.

## Introduction

Nearly 70 years ago, bacterial strains now known as enteropathogenic *Escherichia coli* (EPEC) were first reported to cause neonatal and infant diarrhea with high case-fatality rates [1,2]. During the ensuing years, investigators made great strides in elucidating the molecular mechanisms and cell biology of EPEC infection. EPEC strains use a Type 3 Secretion (T3S) system to inject into host cells a variety of proteins that disrupt the actin cytoskeleton, block innate immune responses, and modulate apoptosis [3,4]. The ability to deliver these effector proteins is essential for disease in humans [5]. Infection of enterocytes leads to loss of microvilli and the formation of cup-like pedestals to which the bacteria intimately adhere, a process known as attaching and effacing [6,7]. Typical EPEC (tEPEC) strains also produce a type IV bundle-forming pilus (BFP) [8], the individual fibers of which intertwine and cause the bacteria to form aggregates that initially attach to cells. Genomic characterization of multiple EPEC strains reveals a complex evolutionary history, evidence of extensive horizontal transfer of critical virulence determinants, and thousands of genes that are not universal among EPEC [9].

Since initial reports, the global epidemiology of EPEC infections has evolved. Whereas in the past, tEPEC strains were reported to be a leading bacterial cause of neonatal diarrhea in numerous developing countries [10–13], their prevalence appears to have declined coincident with socioeconomic gains [14]. However, the recently published results of the Global Enteric Multicenter Study (GEMS), the most comprehensive investigation of the etiology of moderate-to-severe diarrhea (MSD) in childhood yet performed, shed new light on the virulence of tEPEC strains [15]. While tEPEC strains were not responsible for a large burden of disease in the four African and three Asian sites surveyed, they were associated with a significantly elevated risk of mortality in young infants with MSD.

To gain insight into bacterial factors that play a role in severe clinical outcomes of tEPEC infection, we conducted a unique, matched case-control genomic investigation of tEPEC strains from children enrolled in GEMS who had lethal infections (LIs), compared with children who had non-lethal symptomatic infections (NSIs) and asymptomatic infections (AIs).

## Methods

### Strains

Strains were cultured from the feces of infants or children who had acute MSD or who were recruited as healthy controls; tEPEC strains had been identified as described [16]. Individual colonies were shipped from countries of origin to Baltimore, Maryland, USA and verified by PCR, using primers listed in S1 Table as previously described [9] for *bfpA* and *escV* to identify tEPEC, and *stxA1* and *stxA2* to identify and exclude Shiga-toxin producing *E*. *coli*. PCR using the same conditions and primers also listed in S1 Table was used to detect *nleG* clusters in a larger collection of 63 NSI and 56 AI unmatched tEPEC and atypical EPEC (aEPEC, *bfpA-/escV+/stx-*) GEMS isolates.

### Study design

To maximize the probability that observed differences between groups would be due to bacterial genetic factors, each available tEPEC LI strain was matched to EPEC strains from NSI and AI subjects on site and sex, and then proximity-matched using the Mahalanabis method [17] on a range of other variables found to be associated with LI status. Importantly, this matching is completely distinct from the original GEMS study because in the current study all subjects were culture-positive for EPEC. Details are provided in Supporting Information. The strength of these associations was weighed in the Mahalanabis procedure to derive a propensity score. Matches were sought to minimize the propensity score and identify the best possible match. When no match with a tEPEC strain was available, an aEPEC strain was selected by the same criteria.

### Sequencing and analysis

Detailed genomic sequencing and assembly methods are available in Supporting Information. The average genome coverage was approximately 226-fold.

Genes were predicted in each of the 70 genomes and related genes were then grouped with uclust [18] into gene clusters based on the degree of similarity, using an nucleotide identity threshold of 90%. Following the clustering, a file was generated that contained a consensus sequence for each cluster. The consensus sequences were then translated and compared to each genome using TBLASTN [19] as described above. The maximum TBLASTN bit score value obtained for each cluster was used as the denominator to generate a ratio for the cluster compared to each genome. The level of similarity of protein-encoding genes was compared across all 70 genomes in this study using a large-scale BLAST score ratio (LS-BSR) analysis as previously described [20]. The presence and absence of each potential gene cluster in the 70 genomes was ascertained using established thresholds for the BSR analysis [20].

The Stata 12 statistical software package (Statacorp LP, College Station, TX) was used to perform the preparatory logistic regression for the Mahalanobis proximity matching, and the Stata module *mahapick* was used to perform the matching and calculate propensity scores. The significance of associations between particular gene clusters and clinical outcome in matched strain pairs was analyzed using McNemar’s exact test [21], which compares the number of discordant pairs in which the variable is present in the case and absent in the control to the number in which the variable is absent in the case and present in the control. First, all strain pairs were considered and subsequently, to reduce the risk of type I error, subgroups of only well-matched strain pairs and only tEPEC strain pairs were analyzed. Pearson’s chi-square test was used to test the significance of differences in prevalence of particular gene clusters between unmatched NSI and AI strains.

### Ethics statement

The research reported in this study was exempt from institutional review.

## Results

### Strain matching

**Fig 1:**
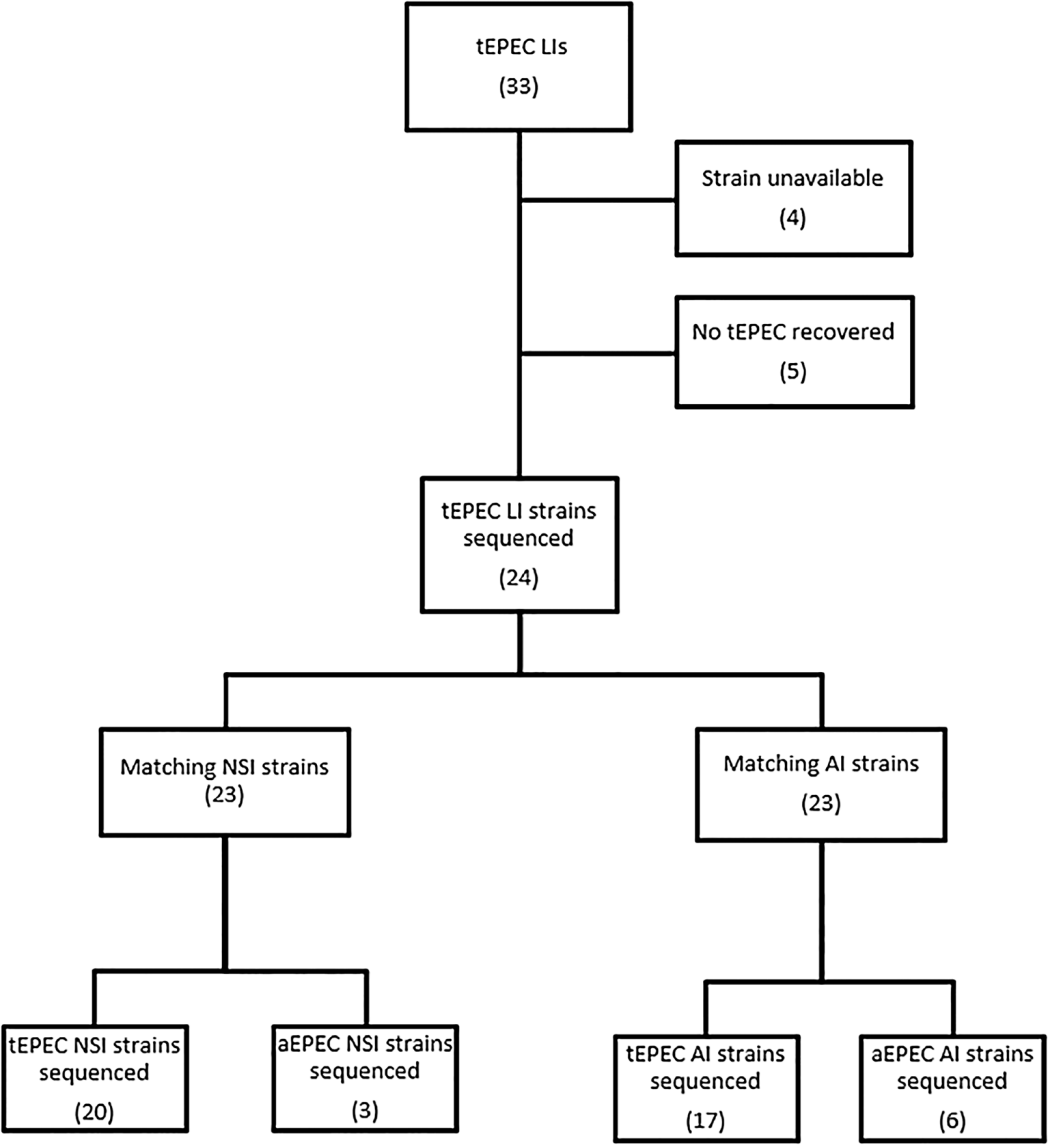
Flow diagram depicting deposition of strains for this study. For each available tEPEC strain from a LI, the closest matching strain from a NSI in a child of the same gender from the same location and from an AI in a child of the same gender from the same location were selected. When no matching tEPEC strain was available, an aEPEC strain with the same constraints was chosen. The same NSI strain served as the control for two LI strains and the same AI strain served as a control for two different LI strains.

Thirty-three infants and children enrolled in GEMS who had had acute MSD and tEPEC identified in the stool did not survive 60 days [15]. tEPEC strains were available and verified from twenty four (73%) of these infants with LI (Fig 1). For each of these LI cases, the most closely matched case with NSI and control with AI, based on propensity score, who were infected with tEPEC (or aEPEC if no tEPEC strain was available), came from the same site and had the same gender, were selected (see Supporting Information for factors affecting propensity score, S2 Table for matching details). A single NSI strain was the closest match for two LI strains and a single AI strain was the closest match for two other LI strains (S2 Table). Thus, 70 strains in total were selected for genomic analysis (Fig 1). NSI patients and LI patients were similar with regard to relevant clinical variables (Table 1). As might be expected, AI controls were not as closely matched (Table 1), particularly for height-for-age Z-score. The only significant difference between the groups was discordance between LI-AI pairs regarding whether or not the bacteria were tEPEC (P = 0.031, McNemar’s exact test), due to the unavailability of tEPEC AI strains to serve as controls for some LI strains. From the distribution of propensity scores, 19 LI-NSI strain pairs and 16 LI-AI strain pairs with scores ≤ 3 were considered well-matched (see Supporting Information).

**Table 1:** Results of propensity matching of strains from lethal infections (LIs), non-lethal symptomatic infections (NSIs), and asymptomatic infections (Ais).

	LI (n = 24)	NSI (n = 23)	AI (n = 23)
Median propensity score (interquartile range)	-	0.2 (0.1–1.1)	1.3 (0.2–6.5)
Number of strain pairs with scores ≤ 3 (percent)	-	19 (79%)	16 (67%)
Median age in months (interquartile range)	8.5 (6–11.25)	7.5 (4–12.5)	7.5 (6–10)
Height-for-age Z-score (mean ± SD)	-2.68 ± 1.87	-2.49 ± 1.32	-1.88 ± 1.25
Partially breast fed (percent)	67%	74%	78%
Exclusively breast fed (percent)	4%	13%	4%
tEPEC (percent)	100%	91%	78%
Presence of co-pathogens (percent)	46%	52%	39%

### Association of genes with severe outcome

We used a large-scale BLAST score ratio (LS-BSR) analysis to examine the gene content of the 70 EPEC isolates. In the LS-BSR analysis, predicted genes from each genome are grouped together into clusters when they have ≥ 90% nucleotide identity (Sahl *et al*., 2013). Conversely, alleles of the same gene are scored as different gene clusters when they have <90% nucleotide identity. For the 70 genomes analyzed in this study, there were 14,412 gene clusters identified. Of this total, 1,316 gene clusters were present in all 70 genomes analyzed based on a conservative BSR value of ≥ 0.8 (S3 Table), representing the conserved EPEC core genome.

Interrogation of the LS-BSR patterns and comparative analysis indicated that no two strains exhibited the same pattern and thus the isolates in this collection were not duplicates and did not result from expansion of single clones. No gene clusters were found exclusively in strains from individuals with any of the three clinical outcomes examined. However, we identified 9 clusters that were significantly more prevalent in LI strains than in matched NSI strains, but not matched AI strains and 286 clusters that were significantly more prevalent in LI strains than in matched AI strains, but not matched NSI strains (Table 2). In addition, 7 clusters were more prevalent in comparisons of LI strains with both NSI and AI strains. These 302 clusters may be considered genes putatively associated with LI. We also identified 16 clusters significantly more prevalent in NSI strains, but not AI strains, than in matched LI strains and 62 clusters significantly more prevalent in AI strains, but not NSI strains, than matched LI strains, as well as 13 more prevalent in both NSI and AI strains than LI strains (Table 2). Thus, 91 gene clusters have putative associations with nonlethal infection. The greater number of clusters identified in comparisons between LI and AI strains than between LI and NSI strains indicates, as expected, that EPEC strains from patients who did not survive differ more from those of control subjects with no symptoms than they do from strains of patients with nonlethal MSD.

**Table 2:** Numbers of gene clusters significantly associated with clinical outcome.

	All Strains		Propensity Score <3^b^		tEPEC Only		Propensity Score <3 and tEPEC only	
	type A^a^	type B	type A	type B	type A	type B	type A	type B
LI vs NSI only	9	16	7	1	5	6	4	3
LI vs AI only	286	62	74	18	71	26	14	5
LI vs NSI and AI								
Total^c^	7	13	1	4	5	9	0	2
LI vs NSI	7	13	0	2	5	8	0	1
LI vs AI	6	12	1	3	1	1	0	1

^a^ Type A indicates the number of gene clusters for which there were significantly (P<0.05, McNemar's exact test) more discordant pairs in which the gene cluster was present in the strain from the child with LI and absent from the child with NSI or AI, type B is the reverse.

^b^ Comparisons of strains from a subgroup of LI and NSI or LI and NI subjects limited to pairs that were closely matched as defined in the text and Supporting Information.

^c^ Some gene clusters were identified as discordant between LI and NSI and between LI and AI only in subgroups but not among all strains.

The lists of all gene clusters significantly associated with clinical outcome in comparisons of LI with NSI, LI with AI, and LI with both NSI and AI are shown in S4–S6 Tables. These tables also display the results of pre-specified subgroup analyses comparing only tEPEC strains, only strains from well-matched pairs, and only tEPEC from well-matched pairs, which are summarized in Table 2. For example, when only tEPEC strains are considered, 14 of the 20 gene clusters that were either more prevalent in LI strains than both NSI and AI strains or *vice versa* remained significant for both comparisons. Additional gene clusters remained significant in comparisons between tEPEC strains from LI and NSI or LI and AI, but not both. Subgroup analyses of strains only from well-matched pairs with propensity scores < 3 reduced the number of significant clusters to five. Similarly, 74 clusters were more prevalent in LI than AI and 18 more prevalent in AI than LI when only well-matched strain pairs were considered, 71 clusters were more prevalent in LI than AI and 26 more prevalent in AI than LI when only tEPEC strains were compared, and 15 were more prevalent in LI than AI and 5 more prevalent in AI than LI when only well-matched pairs with tEPEC were considered. For LI comparisons with NSI, these numbers were 7 and 1, 5 and 6, and 4 and 3, respectively (Table 2). Some clusters were significant only in subgroup analyses (S4–S6 Tables).

### Notable associations of genetic loci and clinical outcome

The largest categories of loci associated with outcome identified encode bacteriophage-related and hypothetical proteins. Several genes that have plausible links to virulence were also overrepresented according to clinical outcome. A gene cluster encoding closely related alleles of *rfbB* was significantly associated with LI, both in comparisons to NSI and LI strains. RfbB, a dTDP-glucose 4,6-dehydratase, is part of the O-specific LPS synthesis pathway (www.ecocyc.org). A gene cluster encoding the Wza polysaccharide capsule transporter protein (www.ecocyc.org) was significantly more prevalent when all LI and matched AI strains were considered, but not when only tEPEC or well-matched strains were compared.

Interestingly, some gene clusters encoding putative virulence genes were significantly associated with non-lethal infection. Clusters encoding the T3S effectors EspJ and OspB were significantly more common in AI than matched LI strains, although significance was not maintained in the pre-specified subgroup analyses limited to well-matched or tEPEC-only strains (S5 Table).

The T3S effector protein NleG, an E3 ubiquitin ligase (Wu *et al*., 2010), shows extensive sequence variation, with 11 *nleG* gene clusters identified in the current collection and many strains having multiple alleles. Against this backdrop emerged a remarkable degree of bias with regard to the presence of certain *nleG* gene clusters among matched isolates from LI and AI subjects (S5 Table). Whereas, in 12 such pairs, cluster 6826 was present in the LI strain, but absent in the AI strain, in only one such pair was this cluster present in the AI strain and absent in the LI strain. Conversely, gene cluster 4759 and gene cluster 6719 were present in the AI strain and absent in the LI strain in six and seven pairs, respectively, whereas in no pairs was either cluster present in the LI strain and absent in the AI strain. Thus, it appears that different *nleG* alleles are strongly associated with the extremes of virulence observed in this study.

### Further investigation of the association of *nleG* clusters with disease

Because of the extremely large number of clusters identified compared to the relatively small number of matched subjects available, correction for multiple comparisons virtually eliminates the potential for statistically significant associations. Therefore, we sought to determine whether an association we identified could be replicated in a larger data set. Ideally, additional matched LI-NSI and LI-AI pairs would be examined; however, we know of no source of additional LI strains. However, 63 additional NSI strains and 56 additional AI strains from GEMS were available for testing. Clusters that were significantly associated, either positively or negatively, with lethal outcome in the LI versus AI analysis (S5 Table), but not the LI versus NSI analysis (S4 Table) or *vice versa*, might reasonably be expected to differ in prevalence between NSI and AI strains. The various *nleG* clusters mentioned above met these criteria as they showed strong discordance in comparisons of matched LI and AI pairs, but no significant association with outcome in matched LI and NSI pairs. Additionally, as the T3S system is linked to virulence, and *nleG* was the only T3S effector both positively and negatively associated with outcome, we decided to focus on this locus. Indeed, *nleG* cluster 6826 was identified by PCR in 43 of 63 (68%) of NSI strains and 28 of 56 (50%) of AI strains (P = 0.043). Conversely, *nleG* cluster 6719 was present in 10 of 63 (16%) NSI strains and 18 of 56 (32%) AI strains (P = 0.037). However, no significant association with outcome was found for *nleG* cluster 4759.

## Discussion

Since the initial identification of tEPEC as a cause of infant diarrhea, it has been appreciated that this infection can be deadly [1,22]. This early observation has been reaffirmed in the modern era [23], most recently by the GEMS, in which isolation of tEPEC among infants 1–11 months of age who have MSD more than doubled the risk of death during the follow-up period [15]. Indeed, 6.4% of such infants did not survive for 60 days. While the cause of death in these infants is uncertain, it is likely that host susceptibility influenced by factors such as passively transferred maternal immunoglobulins, genetic predisposition, degree of malnutrition and concurrent infection conspired with specific bacterial virulence factors to produce a deadly outcome. The relative contribution of genetic variation in bacterial virulence to clinical outcome is unknown.

We used a novel genomic epidemiological approach to test the hypothesis that variation in the presence and absence of genes is associated with lethal outcome in EPEC infection. The average *E*. *coli* genome has approximately 5000 genes, only about 2000 of which are shared among all members of the species [9,24,25]. The balance of genes not only determines the pathovar of the isolate, but dictates a remarkable degree of variation among strains within each pathovar. We reasoned that some of the variability in tEPEC gene content could exert a strong influence on clinical outcome.

To test our hypothesis, we generated draft genome sequences of 70 EPEC strains including 24 tEPEC strains associated with LI, 23 matched strains from children with NSI, and 23 matched strains from children with AI. To minimize the contributions of host factors, we used a propensity-matching method to select the closest available matches for each LI isolate.

Among the 70 genomes analyzed, we identified 14,412 distinct gene clusters, defined as potential protein-coding DNA sequences differing by 10% or more from all other sequences. As expected, many of these clusters were unevenly distributed among LI, NSI and AI strains. Among the 392 gene clusters that showed statistically significant associations with lethality, the majority encodes hypothetical or bacteriophage proteins, the potential contribution to virulence of which is difficult to assess. However, the contribution of several identified bacterial factors to severe clinical outcomes is entirely feasible.

Since the discovery of EPEC, a limited number of O-antigen serogroups has been associated with disease (Kauffmann and Dupont, 1950;Taylor and Charter, 1952). A focus on the O-antigen as a virulence determinant by early investigators was superseded by the identification of specific virulence genes such as those that encode the T3S system and the BFP that now define EPEC [26]. However, the contribution of particular O-antigen types to virulence remains undefined. An *rfbB* gene cluster encoding dTDP-glucose 4,6-dehydratase, an enzyme required for synthesis of O-specific lipopolysaccharide, was significantly associated with LI in comparisons to both NSI and AI strains (S6 Table). Gene clusters most closely related to *wblO* and *wblQ* potentially encoding glucose-1-phosphate thymidylyltransferase and UDP-4-amino-4-deoxy-L-arabinose-oxoglutarate aminotransferase, respectively, had similar associations and may also be involved in O-antigen synthesis. Thus, the findings in this study resurrect the possibility that particular O-antigens or other surface carbohydrates may modulate tEPEC virulence.

The associations of particular *nleG* gene clusters with clinical outcome were particularly noteworthy. We not only identified different *nleG* clusters associated with lethal versus asymptomatic infections and *vice versa*, but were also able to confirm the expected difference in prevalence between additional strains from nonlethal symptomatic versus asymptomatic children for two of these clusters. NleG proteins are E3 ubiquitin ligases, and the remarkable variety of *nleG* genes, including 14 alleles in the Sakai strain of enterohemorrhagic *E*. *coli*, has been previously noted [27]. The cellular targets of NleG proteins have not yet been identified, but it is tempting to speculate that the diversity of NleG proteins is related to substrate specificity, which in turn influences clinical outcome. Further studies to test this hypothesis are indicated.

To our knowledge, this is the first study to use genome sequencing to identify associations between *E*. *coli* variability and clinical outcome, although less comprehensive genetic analyses have been performed in the past. In a study limited to aEPEC strains from children with diarrhea and controls in Norway, the prevalence of 182 virulence genes of various *E*. *coli* pathovars was compared using an oligonucleotide array [28]. Genes from a pathogenicity island including those encoding T3S system effectors NleB, NleE, lymphostatin and EspL were significantly more common in strains from children with diarrhea. Interestingly, those same genes were identified in a completely different context [29]. Seventy-two Shiga toxin producing *E*. *coli* strains were specifically tested for these four genes by PCR. Their prevalence was greater in strains associated with two markers of increased severity: those from outbreaks compared to sporadic cases and those associated with hemolytic-uremic syndrome. Given the large differences in patient populations, strains and methods between these studies and the current study, it should perhaps not be surprising that we did not find associations of these genes with lethal infection in the current study.

Our study has a number of weaknesses, although we know of none that could have been avoided. Although we took great care to optimize matching, differences between LI cases and NSI cases and AI controls persist, particularly with regard to those pairs for which no tEPEC strain was available. The greater heterogeneity of aEPEC in comparison to tEPEC strains [9] adds further genomic complexity, potentially obscuring results. Our pre-specified analysis limited to tEPEC strain pairs is useful in this regard, but at the cost of reducing power to detect differences. Although our collection of 24 tEPEC strains associated with LI is unprecedented in its size, the number of strain pairs studied is relatively small for an epidemiological study, a major factor limiting our ability to detect differences in the prevalence of genes according to clinical outcome. This issue is especially problematic given the large number of statistical comparisons (14,412) made. Accordingly, traditional approaches such as Bonferroni corrections and false discovery rate calculations to limit the possibility of type I statistical errors were impractical, as these methods would have yielded no residual significant associations at a considerable cost of type II errors. These considerations render broad-scale investigations of finer detail differences in our data, such as single-nucleotide polymorphisms or particular alleles of known virulence factors, entirely unrealistic in the absence of specific *a priori* hypotheses. Thus, the associations we identified must be viewed cautiously as requiring further validation.

Our study also has notable strengths, including a highly relevant and objective clinical outcome (mortality), a rigorous matching procedure, the comprehensive detail afforded by highly redundant draft genome sequencing, and the pre-specified subgroup analyses designed to mitigate the effects of imperfect strain matching. Our results are supported by the biological plausibility of some of the loci we identified. Our results are further supported by the expected observation of larger differences in comparisons between LI and AI strains than between LI and NSI strains. Furthermore, differences in the prevalence of two versions of *nleG* in a larger set of strains validated the association found in the initial analysis, adding additional confidence to our conclusions.

This unique analysis of genetic variation among tEPEC strains according to clinical outcome provides valuable information linking specific genes to risk of and protection from lethality. The list of such genes provides fertile grounds for further investigations with the ultimate goal of preventing severe EPEC disease.

## Supporting Information

S1 Methods Detail statistical and genomic methods are presented in this section. (DOCX)

S1 Table Primers used in this study. (DOCX)

S2 Table Genome characteristics of the isolates sequenced in this study and clinical characteristics of the subjects from which they were cultured. (PDF)

S3 Table Prevalence of gene clusters identified using LS-BSR in genomes by clinical outcome. (DOCX)

S4 Table Gene clusters identified more frequently in strains from lethal infections (LIs) than non-lethal symptomatic infections (NSIs) or *vice versa*. (PDF)

S5 Table Gene clusters identified more frequently in strains from lethal infections (LIs) than asymptomatic infections (AIs) or *vice versa*. (PDF)

S6 Table Gene clusters identified more frequently in strains from lethal infections (LIs) than both non-lethal symptomatic infections (NSIs) and asymptomatic infections (AIs) or *vice versa*. (PDF)
