# Supplementary material for: Bacterial Factors Associated with Lethal Outcome of Enteropathogenic Escherichia coli Infection: Genomic Case-Control Studies
Source: PLoS Negl Trop Dis. 2015 May 15;9(5):e0003791. doi: 10.1371/journal.pntd.0003791 (PMC4433268; doi:10.1371/journal.pntd.0003791)
Supplement: S1 Methods — (DOCX) [file pntd.0003791.s001.docx]

**Supplemental Methods**

**Statistical methods.** To maximize the probability that observed differences between groups would be due to bacterial genetic factors, and not due to confounding environmental, socio-demographic or host-specific protective- or risk-factors, each available tEPEC LI strain was matched to NSI and AI strains on site and sex, and then proximity-matched using the Mahalanabis method [[1](#_ENREF_1)] on a range of other variables found to be associated with LI status. Three separate regression analyses were performed. Firstly, LI strains were set against NSI strains in a logistic regression, and factors suspected to be associated with mortality based on original GEMS data [[2](#_ENREF_2)] were explored for association in step-up model selection: site, sex, age, height for age z-score (HAZ) at enrollment, breastfeeding (partial, exclusive and any breastfeeding were examined separately), and socioeconomic status as defined by quintiles of wealth index [[3](#_ENREF_3)]. Interaction between breastfeeding and age was also explored. Data regarding additional important clinical factors that could have impacted mortality, such as HIV status, co-morbidities other than co-infections, and presence of organ system failure, were not collected during GEMS and were therefore not available for investigation. This analysis confirmed that age, enrollment HAZ, partial breastfeeding and exclusive breastfeeding were potential causes of the difference between LI and AI cases, and would therefore be used for proximity matching. Due to the scarcity of data in this sub-study, a separate logistic regression was then performed among cases on the full GEMS dataset, comparing those who did and did not die regardless of whether they were infected with tEPEC, to identify pathogens associated with death, including as covariates those variables found to be important in the previous regression, and adding a shortlist of pathogens that were present among the strains selected for the ERIN study: Adenovirus (non-40/41), *Cryptosporidium parvum*, enteroaggregative *Escherichia coli* (EAEC) harboring the *aatA* gene [EAEC (aatA)], *Salmonella enterica* (serovars other than Typhi), *Shigella flexneri*, *Giardia lamblia*, EAEC harboring the *aaiC* gene [EAEC (aaiC)], enterotoxigenic *E. coli* (ETEC) producing heat-stable toxin but not heat-labile toxin [ETEC (ST only)], *Entamoeba histolytica*, Rotavirus and *Campylobacter coli*. This analysis revealed that *C. parvum*, *S. enterica* (serovars other than Typhi), EAEC (aaiC), ETEC (ST only), Rotavirus and *S. flexneri* were positively associated with mortality among cases, while *G. lamblia* was negatively associated with mortality. These variables were therefore selected for proximity matching, together with age, enrollment HAZ, partial breastfeeding and exclusive breastfeeding, while requiring that sex and age be matched exactly. This procedure was repeated for LI vs NSI, finally selecting age, enrollment HAZ, *C. parvum*, *S. enterica* (serovars other than Typhi), *G. lamblia*, EAEC (aaiC), ETEC (ST only) and *E. histolytica* as proximity matching variables, while also requiring that site and sex be matched exactly. The strength of these associations was weighed in the Mahalanobis procedure to derive a propensity score. Matches were sought to minimize the propensity score. When no match with a tEPEC strain was available, an aEPEC strain lacking the genes for the BFP was selected by the same criteria. A cutoff proximity score of three was selected to define well-matched strains because scores greater than that value were widely separated from one another (Supplemental Table 2).

**Sequencing and analysis.** Genomic DNA was isolated from each strain by growing a single colony overnight in Luria-Bertani (LB) medium at 37˚C with shaking. The genomic DNA was isolated from the overnight culture using the GenElute Genomic kit (Sigma-Aldrich; St. Louis, MO). The genome sequence of each isolate was generated at the Institute for Genome Sciences, Genome Resource Center (http://www.igs.umaryland.edu) on the Illumina HiSeq2000 using paired-end libraries with 300 bp inserts. The draft genomes were assembled using Minimus [[4](#_ENREF_4)] to merge together contigs generated using two different assembly programs. The two assembly algorithms used were the Velvet assembly program [[5](#_ENREF_5)] with kmer values determined using VelvetOptimiser v2.1.4 (http://bioinformatics.net.au/software.velvetoptimiser.shtml), and the Edena v3 assembler [[6](#_ENREF_6)]. The final merged assemblies were filtered to contain contigs ≥ 500 bp. Information on the genome size, the number of contigs and GenBank accession numbers for each strain are listed in Supplemental Table 2.

REFERENCES

1. Mahalanabis PC. On the generalized distance in statistics. ProcNatlInstSciIndia **1936**; 2:49-55.

2. Kotloff KL, Nataro JP, Blackwelder WC, et al. Burden and aetiology of diarrhoeal disease in infants and young children in developing countries (the Global Enteric Multicentre Study, GEMS): a prospective, case-control study. Lancet **2013**; 382:209-22.

3. Filmer D, Pritchett LH. Estimating wealth effects without expenditure data--or tears: an application to educational enrollments in states of India. Demography **2001**; 38:115-32.

4. Sommer DD, Delcher AL, Salzberg SL, Pop M. Minimus: a fast, lightweight genome assembler. BMCBioinformatics **2007**; 8:64.

5. Zerbino DR, Birney E. Velvet: algorithms for de novo short read assembly using de Bruijn graphs. Genome Res **2008**; 18:821-9.

6. Hernandez D, Francois P, Farinelli L, Osteras M, Schrenzel J. De novo bacterial genome sequencing: millions of very short reads assembled on a desktop computer. Genome Res **2008**; 18:802-9.
