# Supplementary material for: Bacterial Factors Associated with Lethal Outcome of Enteropathogenic Escherichia coli Infection: Genomic Case-Control Studies
Source: PLoS Negl Trop Dis. 2015 May 15;9(5):e0003791. doi: 10.1371/journal.pntd.0003791 (PMC4433268; doi:10.1371/journal.pntd.0003791)
Supplement: S1 Table — (DOCX) [file pntd.0003791.s002.docx]

| **Supplemental Table S1.** Primers used in this study | | |  |  |  |
| --- | --- | --- | --- | --- | --- |
|  |  |  |  |  |  |
| Target | Predicted Protein | Forward primer (5'-3') | Reverse primer (5'-3') | Amplicon size (bp) | Source |
| *stx1A* | shiga toxin subunit 1A | TACCTTAGACTTCTCGACTGCAAA | CCGGACACATAGAAGGAAACTC | 778 | Hazen *et al.,* 2013 |
| *stx2A* | shiga toxin subunit 2A | TAATTTATATGTGGCCGGGTTC | CTGTCGCCAGTTATCTGACATT | 575 | Hazen *et al.,* 2013 |
| *escV* | T3SS component | GCGGGTGACATTATTTACTCCT | AGCATCACCGACAGACAATACA | 431 | Hazen *et al.,* 2013 |
| *bfpA* | bundlin, pilin of bundle-forming pilus | TTTCAGAAGTAATGAGCGCAAC | TTCAASGTTGCAAGACTAACACA | 258 | Hazen *et al.,* 2013 |
| *nleG* cluster 6826 | Ubiquitin Ligase T3SS Effector | GTAGCCAATGAGCGCGGAGATATTACAG | ATTGAATAGCCTCAAGCGGGCACT | 286 | This study |
| *nleG* cluster 6719 | Ubiquitin Ligase T3SS Effector | CTTCGTGCTCAAGTTGCAGCCAATG | AGTTGCATCAGAGGGACACTGGAG | 303 | This study |
| *nleG* cluster 4759 | Ubiquitin Ligase T3SS Effector | GCATTGCATTTATCTCCCTACGAGGT | GTTCTCTTGTGAGATTCATGAACGCAGAC | 626 | This study |
|  |  |  |  |  |  |
