# Supplementary material for: Bacterial Factors Associated with Lethal Outcome of Enteropathogenic Escherichia coli Infection: Genomic Case-Control Studies
Source: PLoS Negl Trop Dis. 2015 May 15;9(5):e0003791. doi: 10.1371/journal.pntd.0003791 (PMC4433268; doi:10.1371/journal.pntd.0003791)
Supplement: S2 Table — (PDF) [file pntd.0003791.s003.pdf]

Supplemental Table S2. Genome characteristics of the isolates sequenced in this study and clinical characteristics of the subjects from which they were cultured.

Matched Set	Child ID[a]	Specimen Id[b]	Clinical Outcome[c]	Country	tEPEC	Draft Genome Size (Mb)	No. contigs	Accession Number	Proximity Score	Age (months)	Height-for-age Z score	Gender	Partially Breast Fed	Exclusively Breast Fed	Co-pathogens[d]										
															Adenovirus, non-4041	*Campylobacter coli*	*Cryptosporidium parvum*	EAEC aaiC2	EAEC aatA2	*Entamoeba histolytica*	ETEC St2	*Giardia lamblia*	Rotavirus	*Salmonella* not typhi	*Shigella flexneri*
	101003810	100290	LI	The Gambia	+	5.42	115	JHQV00000000	-	7	-3.89	male	+	-	+	-	-	-	-	-	-	-	-	-	-
1	103006889	102536	NSI	The Gambia	+	5.30	134	JHRV00000000	0.46	10	-3.05	male	+	-	-	-	-	-	-	-	-	-	-	-	-
	104900329	100175	AI	The Gambia	-	4.85	89	JHSR00000000	0.07	9	-3.84	male	+	-	-	-	-	-	-	-	-	-	-	-	-
	101005490	100343	LI	The Gambia	+	5.07	154	JHQW00000000	-	7	-1.04	female	+	-	-	-	-	-	-	-	-	-	-	-	-
2	102010781	102598	NSI	The Gambia	+	5.36	187	JHRW00000000	0.16	3	-1.04	female	-	+	-	-	-	-	-	-	-	-	-	-	-
	101902697	102535	AI	The Gambia	+	4.79	97	JHST00000000	0.08	6	-0.69	female	+	-	-	-	-	-	-	-	-	-	-	-	-
	101006896	100414	LI	The Gambia	+	5.31	116	JHQX00000000	-	17	-3.24	female	+	-	-	-	-	-	-	-	-	-	-	-	-
3	106003721	102929	NSI	The Gambia	-	5.24	147	JHRX00000000	0.44	23	-3.78	female	-	-	-	-	-	-	-	-	-	-	-	-	-
	101902062	102132	AI	The Gambia	-	5.28	110	JHSS00000000	0.76	10	-2.94	female	+	-	-	-	-	-	-	-	-	-	-	-	-
	101023356	102550	LI	The Gambia	+	5.17	209	JHQY00000000	-	17	-1.69	female	-	-	-	-	+	-	+	-	-	-	-	-	-
4	103001671	100329	NSI	The Gambia	+	4.81	390	JHRT00000000	0.65	9	-1.67	female	+	-	-	-	+	-	-	-	-	-	-	-	-
	101904025	103447	AI	The Gambia	+	5.09	182	JHSV00000000	0.39	22	-1.88	female	-	-	-	-	+	-	-	-	-	-	-	-	-
	101039601	103385	LI	The Gambia	+	5.05	149	JHQZ00000000	-	9	-7.73	male	+	-	-	-	-	-	-	-	-	-	-	+	-
5	102007452	100854	NSI	The Gambia	-	4.69	101	JHRU00000000	86.59	15	-3.67	male	+	-	-	-	-	-	-	-	-	-	-	-	-
	101900217	100100	AI	The Gambia	-	5.27	123	JHSQ00000000	71.20	10	-5.62	male	+	-	-	-	-	-	+	-	-	-	-	-	-
	101042580	103573	LI	The Gambia	+	5.15	134	JHRA00000000	-	11	-1.25	female	+	-	-	-	-	-	-	-	-	-	-	-	-
6	101042706	103578	NSI	The Gambia	+	5.11	376	JHRY00000000	0.09	14	-1.36	female	+	-	-	-	-	-	-	-	-	-	-	-	+
	101903875	103338	AI	The Gambia	+	4.66	112	JHSU00000000	0.04	11	-1.51	female	+	-	-	-	-	-	-	-	-	-	-	-	-
	206009697	203741	LI	Mali	+	5.05	446	JHRB00000000	-	7	-1.06	female	+	-	-	-	-	-	-	-	-	-	-	-	-
7	235000568	200146	NSI	Mali	+	5.13	125	JHRZ00000000	0.06	5	-1.25	female	+	-	-	-	-	-	-	-	-	-	-	-	-
	203900028	200077	AI	Mali	+	5.09	141	JHSW00000000	0.07	8	-0.78	female	+	-	-	-	-	-	-	-	-	-	-	-	-
	301000533	300059	LI	Mozambique	+	4.83	122	JHRC00000000	-	12	-2.29	female	+	-	-	-	-	-	-	-	-	-	-	-	-
8	333001672	302150	NSI	Mozambique	+	4.94	286	JHSD00000000	0.09	10	-1.94	female	+	-	-	-	-	-	-	-	-	-	-	-	-
	301903383	303301	AI	Mozambique	+	4.91	100	JHTC00000000	0.39	7	-2.09	female	+	-	-	-	-	-	-	-	-	-	-	-	-
	301000907	300262	LI	Mozambique	+	4.97	111	JHRD00000000	-	10	-2.04	female	-	-	-	-	+	-	-	-	-	+	-	-	-
9	303013978	303145	NSI	Mozambique	+	4.84	102	JHSG00000000	6.91	4	-2.42	female	+	-	-	-	+	-	-	-	-	-	+	-	-
	301900803	300847	AI	Mozambique	+	4.85	385	JHSY00000000	8.85	7	-0.82	female	+	-	-	-	+	-	-	-	-	+	-	-	-
	301038524	302014	LI	Mozambique	+	5.23	527	JHRE00000000	-	16	-2.22	male	-	-	-	-	-	-	-	-	-	-	-	-	-
10	303000196	300231	NSI	Mozambique	+	5.13	79	JHSC00000000	0.47	9	-2.09	male	+	-	-	-	-	-	-	-	-	-	+	-	-
	301900197	300469	AI	Mozambique	+	4.51	64	JHSX00000000	6.19	3	-3.02	male	+	-	-	-	-	-	-	-	-	-	-	-	-
	301043618	302048	LI	Mozambique	+	5.27	436	JHRF00000000	-	8	-3.79	female	+	-	-	-	-	-	-	-	-	-	-	-	-
11	301000624	300075	NSI	Mozambique	+	4.97	103	JHSA00000000	0.21	4	-4.08	female	+	-	-	-	-	-	-	-	-	-	+	-	-
	301903444	303341	AI	Mozambique	-	5.06	86	JHTD00000000	1.29	10	-2.41	female	+	-	-	-	-	-	-	-	-	-	-	-	-
	301044158	302053	LI	Mozambique	+	4.94	420	JHRG00000000	-	11	-3.72	male	+	-	-	-	+	-	-	-	-	-	-	-	-
12	303000127	300214	NSI	Mozambique	+	5.07	104	JHSB00000000	0.79	7	-2.49	male	-	+	-	-	+	-	-	-	-	-	-	-	-
	303900305	302312	AI	Mozambique	+	5.15	115	JHTA00000000	6.97	28	-2.47	male	-	-	-	-	+	-	-	+	-	-	-	-	-
	301052167	302662	LI	Mozambique	+	5.13	478	JHRI00000000	-	1	-3.59	female	+	-	-	-	-	-	-	-	-	-	-	-	-
13	301000624	300075	NSI	Mozambique	+	4.97	103	JHSA00000000	0.18	4	-4.08	female	+	-	-	-	-	-	-	-	-	-	+	-	-
	301903383	303301	AI	Mozambique	+	4.91	100	JHTC00000000	2.16	7	-2.09	female	+	-	-	-	-	-	-	-	-	-	-	-	-
	301071084	303289	LI	Mozambique	+	5.09	144	JHRJ00000000	-	11	-3.39	female	+	-	-	-	-	-	-	-	-	+	+	-	-
14	301056017	302909	NSI	Mozambique	+	4.79	254	JHSF00000000	0.17	7	-3.14	female	+	-	-	-	-	-	-	-	-	+	-	-	-
	303900390	303139	AI	Mozambique	+	4.93	150	JHTB00000000	0.53	6	-2.80	female	+	-	-	-	-	-	-	-	-	+	+	-	-
	333002639	302275	LI	Mozambique	+	5.01	394	JHRH00000000	-	6	0.46	male	-	+	-	-	-	-	-	+	-	-	+	-	-
15	301053204	302687	NSI	Mozambique	+	5.00	394	JHSE00000000	9.63	5	-0.62	male	+	-	-	-	+	-	-	+	-	-	+	-	-
	301901567	302137	AI	Mozambique	-	5.10	44	JHSZ00000000	2.82	8	-1.71	male	-	+	-	-	-	-	-	-	-	-	+	-	-
	404001167	400791	LI	Kenya	+	5.32	170	JHRK00000000	-	6	-0.43	male	+	-	-	+	-	-	-	-	-	-	-	-	-
16	409000811	401091	NSI	Kenya	+	5.09	89	JHSI00000000	0.01	6	-0.28	male	+	-	+	-	-	-	-	-	-	-	-	-	-
	405902300	403341	AI	Kenya	-	5.17	111	JHTK00000000	0.01	6	-0.57	male	+	-	-	-	-	-	-	-	-	-	-	-	-
	405002579	401264	LI	Kenya	+	5.14	228	JHRO00000000	-	1	-0.18	male	+	-	-	-	-	-	-	-	-	-	-	-	-
17	408000165	400738	NSI	Kenya	+	5.16	102	JHSH00000000	0.01	1	-0.05	male	-	+	+	-	-	-	-	-	-	-	-	-	-
	408900628	400929	AI	Kenya	+	5.04	139	JHTE00000000	0.08	3	-0.39	male	+	-	-	-	-	-	+	-	-	-	-	-	-
	405006609	401954	LI	Kenya	+	5.32	287	JHRP00000000	-	11	-4.20	male	+	-	-	-	-	-	-	-	-	-	+	-	-
18	411004466	402310	NSI	Kenya	+	5.11	94	JHSM00000000	0.08	9	-4.45	male	+	-	-	-	-	-	-	-	-	-	-	-	-
	408900255	401195	AI	Kenya	+	4.89	194	JHTF00000000	23.09	7	-3.48	male	+	-	-	-	-	-	-	-	-	-	-	-	-
	405007448	402290	LI	Kenya	+	5.36	109	JHRQ00000000	-	3	-1.09	female	+	-	-	-	-	-	-	-	-	-	-	-	-
19	411006247	402804	NSI	Kenya	+	5.48	135	JHSN00000000	0.05	1	-1.20	female	-	-	-	-	-	-	-	-	-	-	-	-	-
	404900543	401675	AI	Kenya	+	4.95	125	JHTG00000000	0.06	5	-1.11	female	+	-	-	-	-	-	-	-	-	-	-	-	-
	407001314	401140	LI	Kenya	+	5.00	255	JHRM00000000	-	12	-4.57	male	-	-	-	-	-	-	-	-	-	-	-	-	-
20	408001718	401210	NSI	Kenya	-	4.57	781	JHSJ00000000	0.10	15	-4.43	male	+	-	-	-	-	-	-	-	-	-	-	-	-
	408902032	402981	AI	Kenya	+	5.17	75	JHTI00000000	2.19	9	-2.67	male	-	-	-	+	-	-	-	-	-	-	-	-	-
	407001479	401150	LI	Kenya	+	5.08	142	JHRN00000000	-	49	-3.74	female	-	-	-	-	+	-	-	-	-	-	-	-	-
21	404002818	401817	NSI	Kenya	+	4.90	96	JHSL00000000	12.35	43	-1.85	female	-	-	-	-	-	-	-	-	-	-	-	-	-
	405902255	403116	AI	Kenya	+	4.97	108	JHTJ00000000	20.30	39	-1.24	female	-	-	-	-	-	-	-	-	-	-	-	-	-
	411001161	401031	LI	Kenya	+	5.04	170	JHRL00000000	-	6	-4.35	female	+	-	-	-	-	-	-	-	-	-	-	-	-
22	407002750	401588	NSI	Kenya	+	5.10	100	JHSK00000000	1.24	7	-2.75	female	+	-	-	-	-	-	-	-	-	-	-	-	-
	408901063	402559	AI	Kenya	+	4.99	288	JHTH00000000	4.55	5	-1.60	female	+	-	-	-	-	-	-	-	-	-	-	-	-
	701012682	702324	LI	Pakistan	+	5.01	96	JHRR00000000	-	2	-4.53	male	-	-	-	-	+	-	-	+	-	-	-	-	-
23	707022601	702626	NSI	Pakistan	+	5.23	237	JHSP00000000	39.85	12	-4.00	male	-	-	-	-	+	-	-	-	-	-	-	-	-
	702900185	700283	AI	Pakistan	+	4.76	97	JHTL00000000	18.84	6	-1.21	male	+	-	-	-	+	-	-	-	-	-	-	-	-
	702026545	703533	LI	Pakistan	+	5.30	549	JHRS00000000	-	1	-0.83	male	-	-	-	-	-	-	-	-	-	-	-	-	-
24	702012431	702423	NSI	Pakistan	+	5.40	181	JHSO00000000	0.04	3	-0.87	male	+	-	-	-	-	-	+	-	-	-	-	-	-
	702901552	703450	AI	Pakistan	+	5.52	289	JHTM00000000	0.94	8	-0.36	male	-	-	-	-	-	-	-	-	-	-	-	-	-

[a]Note that the strain from child ID 301903383 served as the AI control for both matched set 8 and 13 and the strain from child ID 301000624 served as the NSI control for both matched sets 11 and 13.

[b]The specimen ID is unique for each isolate and genome sequence.
[c]Clinical outcomes are classified as lethal infection (LI), non-lethal symptomatic infection (NSI), and asymptomatic infection (AI).
[d]Presence or absence of co-pathogens also associated with lethal infection in regression analysis. See supplemental information text for more detail.

average	5.06	194
sd	0.20	141
