# Supplementary material for: Bacterial Factors Associated with Lethal Outcome of Enteropathogenic Escherichia coli Infection: Genomic Case-Control Studies
Source: PLoS Negl Trop Dis. 2015 May 15;9(5):e0003791. doi: 10.1371/journal.pntd.0003791 (PMC4433268; doi:10.1371/journal.pntd.0003791)
Supplement: S3 Table — (DOCX) [file pntd.0003791.s004.docx]

| **Supplemental Table S3.** Prevalence of gene clusters identified using LS-BSR in genomes by clinical outcome | | | | | | | |
| --- | --- | --- | --- | --- | --- | --- | --- |
|  |  |  |  |  |  |  |  |
|  |  | No. of Clusters Present | | | | |  |
| Clinical Outcome^a^ | No. Genomes | 100% | ≥80% | ≥50% | <50% | 0 | Exclusive^b^ |
| All | 70 | 1,316 | 4,776 | 5,951 | 8,467 | 0 | - |
| LI | 24 | 2,867 | 4,822 | 6,140 | 8,278 | 2,571 | 0 |
| NSI | 23 | 2,907 | 4,654 | 5,928 | 8,490 | 2,062 | 0 |
| AI | 23 | 2,412 | 4,640 | 5,864 | 8,554 | 1,984 | 0 |
|  |  |  |  |  |  |  |  |
| ^a^Clinical outcomes are classified as lethal infection (LI), non-lethal symptomatic infection (NSI), and asymptomatic infection (AI). | | | | | | | |
| ^b^Exclusive refers to gene clusters detected in 100% of genomes of the specific clinical outcome that were present in no genomes of the other coutcomes. | | | | | | | |
