# Supplementary material for: Bacterial Factors Associated with Lethal Outcome of Enteropathogenic Escherichia coli Infection: Genomic Case-Control Studies
Source: PLoS Negl Trop Dis. 2015 May 15;9(5):e0003791. doi: 10.1371/journal.pntd.0003791 (PMC4433268; doi:10.1371/journal.pntd.0003791)
Supplement: S4 Table — (PDF) [file pntd.0003791.s005.pdf]

Supplemental Table S4. Gene clusters identified more frequently in strains from lethal infections (LIs) than non-lethal symptomatic infections (NSIs) or *vice versa* .

		All Strains			Propensity Score <3[b]			tEPEC Only			Propensity Score <3 and tEPEC only		
**Cluster number**	**Most similar entry in KEGG database[a]**	**type A[c]**	**type B**	**P**	**type A**	**type B**	**P**	**type A**	**type B**	**P**	**type A**	**type B**	**P**
9781	hypothetical protein	10	1	0.012	8	0	0.008	8	1	0.039	6	0	0.031
9796	hypothetical protein	10	1	0.012	8	0	0.008	8	1	0.039	6	0	0.031
9755	hypothetical protein	9	1	0.022	7	0	0.016	8	1	0.039	6	0	0.031
5882	putative late gene regulator Q	8	1	0.039	7	0	0.016	8	1	0.039	7	0	0.016
8990	pinH; DNA invertase from prophage CP-933H	10	1	0.012				8	1	0.039			
9177	prophage E2348_PP2, lambdoid	9	1	0.022	8	0	0.008						
12516	hypothetical protein	6	0	0.031	6	0	0.031						
11567	transposase ORF A (fragment), IS911	6	0	0.031	6	0	0.031						
10137	predicted DNA-invertase	8	1	0.039									
8786	hypothetical phage antitermination protein	1	8	0.039				0	7	0.016	0	6	0.031
8543	ybcQ; hypothetical protein	1	8	0.039				0	7	0.016	0	6	0.031
8789	ybcQ2; putative Q protein	1	8	0.039				0	7	0.016	0	6	0.031
6729	rsxB; electron transport complex protein RnfB; K03616 electron transport complex protein RnfB	0	6	0.031				0	6	0.031			
29	Efa1/LifA-like protein	1	9	0.022				1	9	0.022			
3	integrative element E2348_IE2	1	9	0.022				1	9	0.022			
14218	hypothetical protein	2	10	0.039	1	8	0.039						
313	minor tail protein precursor H	3	13	0.021									
321	predicted tail length tape measure protein	3	13	0.021									
320	phage tail tape measure protein, lambda family	3	12	0.035									
12812	hypothetical protein	1	8	0.039									
14009	hypothetical protein	1	8	0.039									
10196	yeeV; pseudogene	1	8	0.039									
10600	yoeF; hypothetical protein	0	6	0.031									
4497	yphB; uncharacterized protein YphB	0	6	0.031									
3714	yphF; putative sugar transporter subunit: periplasmic-binding component of ABC superfamily	0	6	0.031									

[a]The most similar entry in the Kyoto Encyclopedia of Genes and Genomes (KEGG) database (http://www.genome.jp/kegg/kegg1.html) for each gene cluster is shown.

[b]List of gene clusters identified more frequently in strains from pairs of children with LI and NSI, whose Propensity score indicated that they were closely matched.

[c]Type A indicates the number of discordant pairs in which the gene cluster was present in the strain from the child with LI and absent from the child with NSI, type B is the reverse. P is the P value specified by McNemar's exact test.
