# Supplementary material for: Bacterial Factors Associated with Lethal Outcome of Enteropathogenic Escherichia coli Infection: Genomic Case-Control Studies
Source: PLoS Negl Trop Dis. 2015 May 15;9(5):e0003791. doi: 10.1371/journal.pntd.0003791 (PMC4433268; doi:10.1371/journal.pntd.0003791)
Supplement: S5 Table — (PDF) [file pntd.0003791.s006.pdf]

Supplemental Table S5. Gene clusters identified more frequently in strains from lethal infections (LIs) than asymptomatic infections (AIs) or *vice versa*.

Cluster number	Most similar entry in KEGG database[a]	All Strains			Propensity Score <3[b]			tEPEC Only			Propensity Score <3 and tEPEC only		
		type A[c]	type B	P	type A	type B	P	type A	type B	P	type A	type B	P
10776	putative antirepressor protein	11	0	0.001	9	0	0.004	7	0	0.016	7	0	0.016
5209	putative phage repressor protein	11	0	0.001	9	0	0.004	7	0	0.016	7	0	0.016
8416	hypothetical protein	9	0	0.004	7	0	0.016	7	0	0.016	7	0	0.016
8477	hypothetical protein	9	0	0.004	7	0	0.016	7	0	0.016	7	0	0.016
9997	putative transposase	9	0	0.004	7	0	0.016	7	0	0.016	7	0	0.016
10009	putative transposase	9	0	0.004	7	0	0.016	7	0	0.016	7	0	0.016
9631	pseudogene	11	2	0.023	9	0	0.004				7	0	0.016
9606	hypothetical protein ; K09803 hypothetical protein	9	0	0.004	6	0	0.031	8	0	0.008	6	0	0.031
12990	hypothetical protein ; K07484 transposase	11	1	0.006	8	1	0.039	7	0	0.016	6	0	0.031
11819	integrase	10	2	0.039	7	0	0.016	9	1	0.022	6	0	0.031
13219	putative integrase	10	2	0.039	7	0	0.016	9	1	0.022	6	0	0.031
14415	hypothetical protein	10	2	0.039	6	0	0.031	9	1	0.022	6	0	0.031
13063	dhfrVII; pseudogene	11	1	0.006	7	0	0.016	8	1	0.039	6	0	0.031
10814	lambda phage S protein family protein	11	0	0.001	7	0	0.016	8	0	0.008			
11165	hypothetical protein	11	0	0.001	7	0	0.016	7	0	0.016			
8791	putative tail protein	13	1	0.002	7	0	0.016	8	1	0.039			
6727	putative minor capsid protein	12	1	0.003	7	0	0.016	8	1	0.039			
13120	protein ea22	14	1	0.000	9	1	0.022	8	1	0.039			
2393	phage major capsid protein, HK97 family	10	0	0.002	6	0	0.031	8	0	0.008			
6185	pseudogene	10	0	0.002	6	0	0.031	8	0	0.008			
2498	putative major capsid protein	10	0	0.002	6	0	0.031	8	0	0.008			
9572	putative phage portal protein	10	0	0.002	6	0	0.031	8	0	0.008			
6161	putative prohead protease; K06904	10	0	0.002	6	0	0.031	8	0	0.008			
2327	putative phage portal protein	9	0	0.004	6	0	0.031	7	0	0.016			
844	putative mating pair stabilization outer membrane protein TraN precursor; K12058 conjugal transfer mating pair stabilization protein TraN	10	0	0.002	6	0	0.031	6	0	0.031			
9219	tnpM; transposition modulator TnpM	9	0	0.004	6	0	0.031	6	0	0.031			
71	yehH, dinO, ECK2108, JW2102, JW5915, JW5916, molR, sosF, yehF, yehG; pseudogene	7	0	0.016	6	0	0.031	6	0	0.031			
8992	hypothetical protein	11	1	0.006	8	1	0.039	9	1	0.022			
8557	hypothetical protein	12	2	0.013	8	1	0.039	8	1	0.039			
4502	hypothetical protein	10	2	0.039				8	0	0.008			
10620	prophage E2348_PP2, lambdoid	12	2	0.013				10	1	0.012			
14403	hypothetical protein	12	1	0.003				9	1	0.022			
1906	fadL; long-chain fatty acid outer membrane transporter ; K06076 long-chain fatty acid transport protein	11	2	0.023				9	1	0.022			
1242	hypothetical protein	10	1	0.012				6	0	0.031			
13110	transposase	8	1	0.039				6	0	0.031			
9356	hypothetical protein	7	0	0.016				6	0	0.031			
11984	conserved hypothetical protein from bacteriophage origin	6	0	0.031				6	0	0.031			
11716	DNA repair protein, RadC family	6	0	0.031				6	0	0.031			
7597	hypothetical protein	6	0	0.031				6	0	0.031			
7635	hypothetical protein	6	0	0.031				6	0	0.031			
7795	hypothetical protein	6	0	0.031				6	0	0.031			
7877	hypothetical protein	6	0	0.031				6	0	0.031			
8770	hypothetical protein	6	0	0.031				6	0	0.031			
8772	hypothetical protein	6	0	0.031				6	0	0.031			
9758	hypothetical protein	6	0	0.031				6	0	0.031			
10231	hypothetical protein	6	0	0.031				6	0	0.031			
10642	hypothetical protein	6	0	0.031				6	0	0.031			
10884	hypothetical protein	6	0	0.031				6	0	0.031			
13848	hypothetical protein	6	0	0.031				6	0	0.031			
14243	integrative element E2348_IE2	6	0	0.031				6	0	0.031			
12639	putative DNA repair protein	6	0	0.031				6	0	0.031			
8732	yeeU; antitoxin of the YeeV-YeeU toxin-antitoxin system; CP4-44 prophage	6	0	0.031				6	0	0.031			
11576	hypothetical protein	12	3	0.035				10	2	0.039			
8958	HNH endonuclease	11	1	0.006				8	1	0.039			
8968	HNH endonuclease	11	1	0.006				8	1	0.039			
5403	ISEc12 putative ATP-binding protein	11	1	0.006				8	1	0.039			
8970	putative DNase	11	1	0.006				8	1	0.039			

1310	putative transposase ORF1, IS21 family	11	1	0.006				8	1	0.039
12693	haemolysin expression modulating protein Hha-homolog	11	2	0.023				8	1	0.039
12678	pseudogene	11	2	0.023				8	1	0.039
8942	HNH endonuclease	10	1	0.012				8	1	0.039
9055	hypothetical protein	10	1	0.012				8	1	0.039
11061	putative bacteriophage protein	10	1	0.012				8	1	0.039
11339	putative phage portal protein	10	1	0.012				8	1	0.039
11421	ymfR; conserved hypothetical protein; E14 prophage	10	1	0.012				8	1	0.039
7274	hypothetical protein	9	1	0.022				8	1	0.039
7558	hypothetical protein	9	1	0.022				8	1	0.039
7579	hypothetical protein	9	1	0.022				8	1	0.039
9941	hypothetical protein	9	1	0.022				8	1	0.039
10565	hypothetical protein	9	1	0.022				8	1	0.039
12093	hypothetical protein	9	1	0.022				8	1	0.039
7564	yeeW; hypothetical protein	9	1	0.022				8	1	0.039
1196	B; portal protein; minor capsid protein [contains: protein B*] from bacteriophage origin	11	2	0.023	7	0	0.016			
11712	integrative element E2348_IE6	9	0	0.004	7	0	0.016			
6826	T3SS secreted effector NleG-like protein	12	1	0.003	9	1	0.022			
10621	traV; conjugal transfer protein TraV	10	1	0.012	6	0	0.031			
3274	major capsid protein E	10	2	0.039	6	0	0.031			
9401	predicted plasmid partition protein	10	2	0.039	6	0	0.031			
8461	putative DNA packaging protein	10	2	0.039	6	0	0.031			
1986	putative head protein/prohead protease	10	2	0.039	6	0	0.031			
9146	putative head-DNA stabilization protein	10	2	0.039	6	0	0.031			
8936	putative head-tail adaptor	10	2	0.039	6	0	0.031			
8720	putative minor capsid protein	10	2	0.039	6	0	0.031			
9394	putative plasmid partition protein	10	2	0.039	6	0	0.031			
13703	putative portal protein	10	2	0.039	6	0	0.031			
9096	hypothetical protein	9	0	0.004	6	0	0.031			
7158	hypothetical protein; K10352 myosin heavy chain	9	0	0.004	6	0	0.031			
2969	putative late gene regulator; K06905	9	0	0.004	6	0	0.031			
14326	truncated integrase	9	0	0.004	6	0	0.031			
3197	intI; integrase/recombinase	9	1	0.022	6	0	0.031			
4726	dihydropteroate synthase ; K00796 dihydropteroate synthase [EC:2.5.1.15]	8	1	0.039	6	0	0.031			
10798	essD; putative phage lysis protein S; DLP12 prophage	8	1	0.039	6	0	0.031			
10864	traC; conjugal transfer ATP-binding protein TraC; K12063 conjugal transfer ATP-binding protein TraC	8	1	0.039	6	0	0.031			
13441	traC; conjugal transfer ATP-binding protein TraC; K12063 conjugal transfer ATP-binding protein TraC	8	1	0.039	6	0	0.031			
10640	traR; conjugal transfer protein TraR	8	1	0.039	6	0	0.031			
12105	pseudogene	6	0	0.031	6	0	0.031			
8698	hypothetical protein	12	1	0.003	8	1	0.039			
12128	hypothetical protein	14	4	0.031						
12570	conserved predicted protein	11	2	0.023						
12271	hypothetical protein	11	2	0.023						
13365	hypothetical protein	11	2	0.023						
10897	yejO; pseudogene	11	2	0.023						
10964	hypothetical protein	10	1	0.012						
6701	hypothetical protein	10	2	0.039						
7864	hypothetical protein	10	2	0.039						
11105	hypothetical protein	10	2	0.039						
12015	hypothetical protein	10	2	0.039						
14188	hypothetical protein	10	2	0.039						
11098	minor tail protein Z	10	2	0.039						
284	mngB; alpha-mannosidase	10	2	0.039						
8668	pseudogene	10	2	0.039						
13206	putative 3-phenylpropionic acid transporter ; K05820 MFS transporter, PPP family, 3-phenylpropionic acid transporter	10	2	0.039						
8351	putative bacteriophage protein	10	2	0.039						
8367	putative phage head-tail adaptor	10	2	0.039						
971	putative phage terminase	10	2	0.039						
12472	putative phage terminase	10	2	0.039						
11908	putative phage terminase, small subunit	10	2	0.039						
7519	putative phage terminase, small subunit, P27 family	10	2	0.039						
7825	rzpD; putative murein endopeptidase; DLP12 prophage	10	2	0.039						
7830	rzpD; putative murein endopeptidase; DLP12 prophage	10	2	0.039						

4179	ybl124; hypothetical protein	10	2	0.039
8654	yis1; IS600 ORF1-like protein	10	2	0.039
10472	putative tail protein	9	0	0.004
12767	putative tail protein	9	0	0.004
2600	putative tail sheath protein; K06907	9	0	0.004
7390	putative tail tube protein	9	0	0.004
10133	repA2; negative regulator of repA1 expression	9	0	0.004
11886	helix-turn-helix domain protein	9	1	0.022
5739	hypothetical protein	9	1	0.022
5743	hypothetical protein	9	1	0.022
5756	hypothetical protein	9	1	0.022
8361	hypothetical protein	9	1	0.022
9270	hypothetical protein	9	1	0.022
9342	hypothetical protein	9	1	0.022
9357	hypothetical protein	9	1	0.022
9708	hypothetical protein	9	1	0.022
10279	hypothetical protein	9	1	0.022
10462	hypothetical protein	9	1	0.022
10543	hypothetical protein	9	1	0.022
10601	hypothetical protein	9	1	0.022
10618	hypothetical protein	9	1	0.022
10854	hypothetical protein	9	1	0.022
10943	hypothetical protein	9	1	0.022
11167	hypothetical protein	9	1	0.022
12008	hypothetical protein	9	1	0.022
12304	hypothetical protein	9	1	0.022
12413	hypothetical protein	9	1	0.022
12454	hypothetical protein	9	1	0.022
12820	hypothetical protein	9	1	0.022
12933	hypothetical protein	9	1	0.022
13356	hypothetical protein	9	1	0.022
14305	hypothetical protein	9	1	0.022
1706	pseudogene	9	1	0.022
6911	pseudogene	9	1	0.022
9383	pseudogene	9	1	0.022
12254	pseudogene	9	1	0.022
12669	pseudogene	9	1	0.022
12249	putative bacteriophage protein (gene 65)	9	1	0.022
1704	putative phage helicase ; K02314 replicative DNA helicase [EC:3.6.1.-]	9	1	0.022
3890	putative plasmid partition protein	9	1	0.022
11334	hypothetical protein	8	0	0.008
13995	conserved hypothetical protein	8	1	0.039
14141	human down-regulated in multiple cancers-1 homolog 1	8	1	0.039
4683	hypothetical protein	8	1	0.039
4691	hypothetical protein	8	1	0.039
4699	hypothetical protein	8	1	0.039
4710	hypothetical protein	8	1	0.039
6738	hypothetical protein	8	1	0.039
8482	hypothetical protein	8	1	0.039
8715	hypothetical protein	8	1	0.039
8953	hypothetical protein	8	1	0.039
10141	hypothetical protein	8	1	0.039
10177	hypothetical protein	8	1	0.039
11481	hypothetical protein	8	1	0.039
14187	hypothetical protein	8	1	0.039
9699	hypothetical protein ; K06218 RelE protein	8	1	0.039
9717	hypothetical protein ; K06218 RelE protein	8	1	0.039
8694	integrative element E2348_IE2	8	1	0.039
12633	integrative element E2348_IE2	8	1	0.039
13519	integrative element E2348_IE2	8	1	0.039
9853	IS66 family element, transposase	8	1	0.039
10446	IS66 family orf1	8	1	0.039
11310	PII uridylyl-transferase (EC:2.7.7.59); K00990 [protein-PII] uridylyltransferase [EC:2.7.7.59]	8	1	0.039
405	predicted phage tail tape measure protein	8	1	0.039

10049	prophage E2348_PP4, lambdoid	8	1	0.039
11823	pseudogene	8	1	0.039
12014	pseudogene	8	1	0.039
7794	putative endopeptidase ; K01423 [EC:3.4.-.-]	8	1	0.039
10194	putative plasmid stability protein	8	1	0.039
9712	putative plasmid stability protein; K06218 RelE protein	8	1	0.039
9292	putative tail assembly protein of prophage CP-933T; K06905	8	1	0.039
8531	trbF; conjugal transfer protein TrbF	8	1	0.039
8542	trbF; hypothetical protein	8	1	0.039
8632	yeeV; toxin of the YeeV-YeeU toxin-antitoxin system	8	1	0.039
2481	bfpC; hypothetical protein	7	0	0.016
14225	GH24153 gene product from transcript GH24153-RA ; K11292 transcription elongation factor SPT6	7	0	0.016
11703	GK16393 gene product from transcript GK16393-RA	7	0	0.016
833	hypothetical protein	7	0	0.016
1254	hypothetical protein	7	0	0.016
2607	hypothetical protein	7	0	0.016
3431	hypothetical protein	7	0	0.016
3456	hypothetical protein	7	0	0.016
5148	hypothetical protein	7	0	0.016
5695	hypothetical protein	7	0	0.016
7171	hypothetical protein	7	0	0.016
8385	hypothetical protein	7	0	0.016
8701	hypothetical protein	7	0	0.016
8834	hypothetical protein	7	0	0.016
9099	hypothetical protein	7	0	0.016
9706	hypothetical protein	7	0	0.016
9829	hypothetical protein	7	0	0.016
9835	hypothetical protein	7	0	0.016
9839	hypothetical protein	7	0	0.016
10298	hypothetical protein	7	0	0.016
10312	hypothetical protein	7	0	0.016
10985	hypothetical protein	7	0	0.016
12255	hypothetical protein	7	0	0.016
12650	hypothetical protein	7	0	0.016
12818	hypothetical protein	7	0	0.016
14075	hypothetical protein	7	0	0.016
14202	hypothetical protein	7	0	0.016
14221	hypothetical protein	7	0	0.016
9363	hypothetical protein ; K00166 2-oxoisovalerate dehydrogenase E1 component, alpha subunit [EC:1.2.4.4]	7	0	0.016
9790	hypothetical protein ; K02355 elongation factor EF-G [EC:3.6.5.3]	7	0	0.016
8437	hypothetical protein ; K07062	7	0	0.016
1933	integrase	7	0	0.016
10066	ogr; prophage late promoter activator protein	7	0	0.016
14165	ogr; prophage late promoter activator protein	7	0	0.016
8943	phage baseplate assembly protein ; K06903	7	0	0.016
4751	phage capsid scaffolding protein	7	0	0.016
11048	phage tail protein	7	0	0.016
10491	plasmid maintenance protein VagC-homolog	7	0	0.016
14184	prophage E2348_PP7, P2-like	7	0	0.016
12260	pseudogene	7	0	0.016
13068	pseudogene	7	0	0.016
13314	pseudogene	7	0	0.016
4296	putative baseplate assembly protein	7	0	0.016
8520	putative endolysin	7	0	0.016
9254	putative holin	7	0	0.016
3266	putative major capsid protein	7	0	0.016
14351	putative phage regulator	7	0	0.016
3009	putative portal protein	7	0	0.016
13166	putative regulatory protein	7	0	0.016
7239	putative tail formation protein	7	0	0.016
164	putative tail length tape measure protein	7	0	0.016
945	putative terminase large subunit	7	0	0.016
5002	putative terminase small subunit	7	0	0.016
6215	tail completion phage protein	7	0	0.016

7776	tail completion phage protein	7	0	0.016									
5518	transposase	7	0	0.016									
5111	vagD; hypothetical protein	7	0	0.016									
10477	vagD; hypothetical protein	7	0	0.016									
12743	wbdK; putative pyridoxamine 5-phosphate-dependent dehydrase; K12452 CDP-6-deoxy-D-xylo-4-hexulose-3-dehydrase	7	0	0.016									
6656	bfpA; major pilin structural unit bundlin; K12810 major structural subunit of bundle-forming pilus	6	0	0.031									
12176	bfpA; major pilin structural unit bundlin; K12810 major structural subunit of bundle-forming pilus	6	0	0.031									
3636	bfpF; nucleotide binding protein; K02669 twitching motility protein PilT	6	0	0.031									
8627	capsular polysaccharide transport protein ; K01991 polysaccharide export outer membrane protein	6	0	0.031									
683	cytosolic protein	6	0	0.031									
8567	DNA repair protein	6	0	0.031									
8569	DNA repair protein	6	0	0.031									
940	fliC; flagellar filament structural protein (flagellin); K02406 flagellin	6	0	0.031									
11332	GK20821 gene product from transcript GK20821-RA	6	0	0.031									
3192	glycosyl transferase, group 1	6	0	0.031									
5869	hypothetical protein	6	0	0.031									
8206	hypothetical protein	6	0	0.031									
9202	hypothetical protein	6	0	0.031									
9210	hypothetical protein	6	0	0.031									
9297	hypothetical protein	6	0	0.031									
10832	hypothetical protein	6	0	0.031									
11403	hypothetical protein	6	0	0.031									
14107	hypothetical protein	6	0	0.031									
7832	hypothetical protein ; K01423 [EC:3.4.-.-]	6	0	0.031									
9321	P27 family phage terminase small subunit	6	0	0.031									
10380	pseudogene	6	0	0.031									
11230	pseudogene	6	0	0.031									
14410	pseudogene	6	0	0.031									
2722	putative outer membrane pore protein	6	0	0.031									
938	putative phage terminase, large subunit	6	0	0.031									
1406	putative tail length tape measure protein from putative prophage	6	0	0.031									
9002	putative transposase ORF 2, IS66 family	6	0	0.031									
12349	SF4520	6	0	0.031									
2695	wbdK; putative pyridoxamine 5-phosphate-dependent dehydrase; K12452 CDP-6-deoxy-D-xylo-4-hexulose-3-dehydrase	6	0	0.031									
2710	wbdK; putative pyridoxamine 5-phosphate-dependent dehydrase; K12452 CDP-6-deoxy-D-xylo-4-hexulose-3-dehydrase	6	0	0.031									
5279	wza; putative polysaccharide export protein	6	0	0.031									
1557	ygfU; transporter	6	0	0.031									
8440	yscX; hypothetical protein; K04059 type III secretion protein SctX	6	0	0.031									
12138	traT; conjugal transfer surface exclusion protein TraT				7	0	0.016						
2581	putative integrase				6	0	0.031				6	0	0.031
11749	hypothetical protein	1	10	0.012	1	9	0.022	1	9	0.022	1	9	0.022
6556	putative phage repressor protein	0	11	0.001	0	7	0.016	0	8	0.008	0	6	0.031
8537	T3SS component	1	8	0.039	0	6	0.031	1	8	0.039	0	6	0.031
6140	T3SS component	1	8	0.039	0	6	0.031				0	6	0.031
14265	pseudogene	0	9	0.004	0	6	0.031	0	7	0.016			
14067	T3SS secreted effector EspJ-homolog	0	8	0.008	0	6	0.031	0	7	0.016			
13667	hypothetical protein	1	9	0.022	0	6	0.031	0	7	0.016			
13834	pseudogene	1	10	0.012	0	6	0.031	1	8	0.039			
2358	putative inner membrane protein	2	10	0.039	2	6	0.039	1	9	0.022			
6896	type 1 fimbriae regulatory protein FimX ; K07357 type 1 fimbriae regulatory protein FimB	1	8	0.039	1	5	0.039	0	6	0.031			
7922	conserved hypothetical protein from phage origin, putative ATPases with chaperone activity, ATP-binding subunit (COG0542)	2	12	0.013				0	9	0.004			
9315	hypothetical protein	2	12	0.013				0	9	0.004			
9711	hypothetical protein	2	12	0.013				0	9	0.004			
9988	hypothetical protein	2	12	0.013				0	9	0.004			
10318	hypothetical protein	2	12	0.013				0	9	0.004			
12455	hypothetical protein	2	12	0.013				0	9	0.004			
12477	hypothetical protein	2	12	0.013				0	9	0.004			
12959	polarity suppression protein	2	12	0.013				0	9	0.004			
7482	putative transactivation protein	2	12	0.013				0	9	0.004			
12215	CzcA family heavy metal efflux protein ; K07239 heavy-metal exporter, HME family	2	11	0.023				0	8	0.008			

5538	hypothetical protein	2	11	0.023				0	8	0.008			
14174	hypothetical protein	2	10	0.039				0	8	0.008			
9952	escS; T3SS structure protein EscS; K03227 type III secretion protein SctS	0	7	0.016				0	7	0.016			
6719	T3SS secreted effector NleG-homolog	0	7	0.016				0	7	0.016			
11195	escT; T3SS structure protein EscT; K03228 type III secretion protein SctT	1	9	0.022				1	9	0.022			
6785	hypothetical protein	3	12	0.035				1	9	0.022			
2272	putative nucleoside transporter	0	6	0.031				0	6	0.031			
9560	hypothetical protein	1	10	0.012	0	7	0.016						
476	ycjT; pseudogene	1	9	0.022	1	5	0.022						
380	ycjT; putative hydrolase	1	9	0.022	1	5	0.022						
4482	glucose-1-phosphate thymidylyltransferase (EC:2.7.7.24); K00973 glucose-1-phosphate thymidylyltransferase [EC:2.7.7.24]	2	10	0.039	1	6	0.039						
13600	ygjI; pseudogene	2	10	0.039	1	6	0.039						
298	putative tail component of prophage CP-933O	3	12	0.035	1	8	0.039						
4483	T3SS secreted effector OspB-homolog	1	8	0.039	1	6	0.039						
4491	T3SS secreted effector OspB-like protein	1	8	0.039	1	6	0.039						
13165	yjgL; pseudogene	1	11	0.006									
13146	molR; molybdate metabolism regulator, first fragment	2	12	0.013									
14268	hypothetical protein	0	7	0.016									
5042	putative transcription regulator; LuxR-type HTH domain	1	9	0.022									
4529	rmlA; putative glucose-1-phosphate thymidylyltransferase; K00973 glucose-1-phosphate thymidylyltransferase [EC:2.7.7.24]	1	9	0.022									
1707	DNA primase	0	6	0.031									
827	dnaX; DNA polymerase III subunit tau ; K02343 DNA polymerase III subunit gamma/tau [EC:2.7.7.7]	0	6	0.031									
2596	hypothetical protein	0	6	0.031									
9240	hypothetical protein	0	6	0.031									
10432	hypothetical protein	0	6	0.031									
10676	hypothetical protein	0	6	0.031									
10830	hypothetical protein	0	6	0.031									
13429	hypothetical protein	0	6	0.031									
13527	hypothetical protein	0	6	0.031									
2541	integrase family protein	0	6	0.031									
7481	nudD; GDP-mannose mannosyl hydrolase (EC:1.1.1.271); K03207 colanic acid biosynthesis protein WcaH [EC:3.6.1.-]	0	6	0.031									
2079	phage integrase family protein	0	6	0.031									
2555	phage integrase family protein	0	6	0.031									
2560	prophage cp4-57 integrase	0	6	0.031									
4759	T3SS secreted effector NleG-like protein	0	6	0.031									
7477	yffI; putative ethanolaminosome structural protein with putative role in ethanolamine utilization; K04025 ethanolamine utilization protein EutK	0	6	0.031									
14170	ygjI; pseudogene	0	6	0.031									
671	ypfI; predicted hydrolase; K06957	0	6	0.031									
13743	hypothetical protein	3	12	0.035									
3366	escU; T3SS structure protein EscU; K03229 type III secretion protein SctU	1	8	0.039									
7226	fimF; minor component of type 1 fimbriae; K07348 minor fimbrial subunit	1	8	0.039									
10051	hypothetical protein	1	8	0.039									
11520	hypothetical protein										0	6	0.031
8957	chpB; toxin ChpB of the ChpB-ChpS toxin-antitoxin system; K07171				0	6	0.031						
10178	chpS; antitoxin ChpS of the ChpB-ChpS toxin-antitoxin system				0	6	0.031						
7945	hypothetical protein				6	0	0.031						
9365	hypothetical protein				6	0	0.031						
10561	hypothetical protein				6	0	0.031						
11516	hypothetical protein				6	0	0.031						
11976	hypothetical protein				6	0	0.031						
12292	hypothetical protein				0	6	0.031						
12783	hypothetical protein				6	0	0.031						
13925	hypothetical protein				6	0	0.031						
11377	inner membrane protein				6	0	0.031						
10834	IS21 ORF2				6	0	0.031						
12323	Pla2r1, Mrc2_predicted; phospholipase A2 receptor 1; K06560 mannose receptor, C type				6	0	0.031						
10924	pseudogene				6	0	0.031						
11575	putative conjugal transfer protein TraW precursor; K12061 conjugal transfer pilus assembly protein TraW				6	0	0.031						
6659	putative conjugal transfer protein TrbB				6	0	0.031						
2203	putative integrase				6	0	0.031						

283	traC; conjugal transfer ATP-binding protein TraC; K12063 conjugal transfer ATP-binding protein TraC		6	0	0.031
308	traC; conjugal transfer ATP-binding protein TraC; K12063 conjugal transfer ATP-binding protein TraC		6	0	0.031
8586	trbI; conjugal transfer protein TrbI		6	0	0.031
9372	trbJ; conjugal transfer protein TrbJ		6	0	0.031

[a]The most similar entry in the Kyoto Encyclopedia of Genes and Genomes (KEGG) database (http://www.genome.jp/kegg/kegg1.html) for each gene cluster is shown.

[b]List of gene clusters identified more frequently in strains from pairs of children with LI and AI, whose Propensity score indicated that they were closely matched.

[c]Type A indicates the number of discordant pairs in which the gene cluster was present in the strain from the child with LI and absent from the child with AI, type B is the reverse. P is the P value specified by McNemar's exact test.
