# Supplementary material for: Bacterial Factors Associated with Lethal Outcome of Enteropathogenic Escherichia coli Infection: Genomic Case-Control Studies
Source: PLoS Negl Trop Dis. 2015 May 15;9(5):e0003791. doi: 10.1371/journal.pntd.0003791 (PMC4433268; doi:10.1371/journal.pntd.0003791)
Supplement: S6 Table — (PDF) [file pntd.0003791.s007.pdf]

Supplemental Table S6. Gene clusters identified more frequently in strains from lethal infections (LIs) than both non-lethal symptomatic infections (NSIs) and asymptomatic infections (AIs) or *vice versa* .

		LI vs NSI												LI vs AI											
		All Strains			Propensity Score <3[b]			tEPEC Only			Propensity Score <3			All Strains			Propensity Score <3			tEPEC Only			Propensity Score <3		
Cluster number	Most similar entry in KEGG database[a]	type A[c]	type B	P	type A	type B	P	type A	type B	P	type A	type B	P	type A	type B	P	type A	type B	P	type A	type B	P	type A	type B	P
4775	mcrA; 5-methylcytosine-specific restriction endonuclease B; K07451 5-methylcytosine-specific restriction enzyme A [EC:3.1.21.-]	8	1	0.039				8	1	0.039				8	1	0.039				7	0	0.016			
3234	rfbB; dTDP-glucose 4,6 dehydratase ; K01710 dTDP-glucose 4,6-dehydratase [EC:4.2.1.46]	6	0	0.031				6	0	0.031				6	0	0.031									
3449	wblQ; WblQ protein	6	0	0.031				6	0	0.031				6	0	0.031									
4915	wblO; WblO protein ; K00973 glucose-1-phosphate thymidylyltransferase [EC:2.7.7.24]	6	0	0.031				6	0	0.031				6	0	0.031									
8479	WxcM domain-containing protein	6	0	0.031				6	0	0.031				6	0	0.031									
223	putative replication protein for prophage CP-933T	8	1	0.039										8	1	0.039									
6546	predicted DNA invertase	6	0	0.031													6	0	0.031						
12088	hypothetical protein	1	8	0.039	0	6	0.039							2	12	0.013	1	8	0.039	1	11	0.006	1	8	0.039
13469	fimH; protein FimH ; K07350 minor fimbrial subunit	1	8	0.039	0	7	0.016	1	8	0.039	0	7	0.016	1	9	0.022									
314	putative tail length tape measure protein	3	14	0.013				3	12	0.035				2	13	0.007	1	8	0.039						
842	hscA; chaperone protein HscA ; K04044 molecular chaperone HscA	0	7	0.016				0	6	0.016				0	7	0.016									
9873	GpE; tail protein E (GpE)	1	10	0.012				1	9	0.022				1	8	0.039									
657	yraM; uncharacterized protein YraM ; K07121	0	6	0.031				0	6	0.031				0	6	0.031									
6696	dcd; deoxycytidine triphosphate deaminase (EC:3.5.4.13); K01494 dCTP deaminase [EC:3.5.4.13]	0	6	0.031				0	6	0.031				0	6	0.031									
11664	hypothetical protein	1	8	0.039				1	8	0.039				2	10	0.039									
12391	head completion/stabilization protein (GpL)	1	8	0.039				1	8	0.039				2	10	0.039									
11333	prophage E2348_PP6, lambdoid	1	8	0.039													0	6	0.031						
315	putative tail length tape measure protein	4	14	0.031										3	12	0.035									
750	nagE; N-acetyl glucosamine specific PTS system components IIABC; K02802 PTS system, N-acetylglucosamine-specific IIA component [EC:2.7.1.69]; K02803 PTS system, N-acetylglucosamine-specific IIB component [EC:2.7.1.69]; K02804 PTS system, N-acetylglucosamine-specific IIC component	0	6	0.031										0	6	0.031									
2138	csiE; stationary phase inducible protein CsiE	0	6	0.031										0	6	0.031									

[a]The most similar entry in the Kyoto Encyclopedia of Genes and Genomes (KEGG) database (http://www.genome.jp/kegg/kegg1.html) for each gene cluster is shown.

[b]List of gene clusters identified more frequently in strains from pairs of children with LI and NSI, whose Propensity score indicated that they were closely matched.

[c]Type A indicates the number of discordant pairs in which the gene cluster was present in the strain from the child with LI and absent from both the child with AI and the child with NSI, type B is the reverse. P is the P value specified by McNemar's exact test.
